# Supplementary figures and images for: Effect of renin-angiotensin-aldosterone system inhibitors on Covid-19 patients in Korea
Source: PLoS One. 2021 Mar 11;16(3):e0248058. doi: 10.1371/journal.pone.0248058 (PMC7951918; doi:10.1371/journal.pone.0248058)

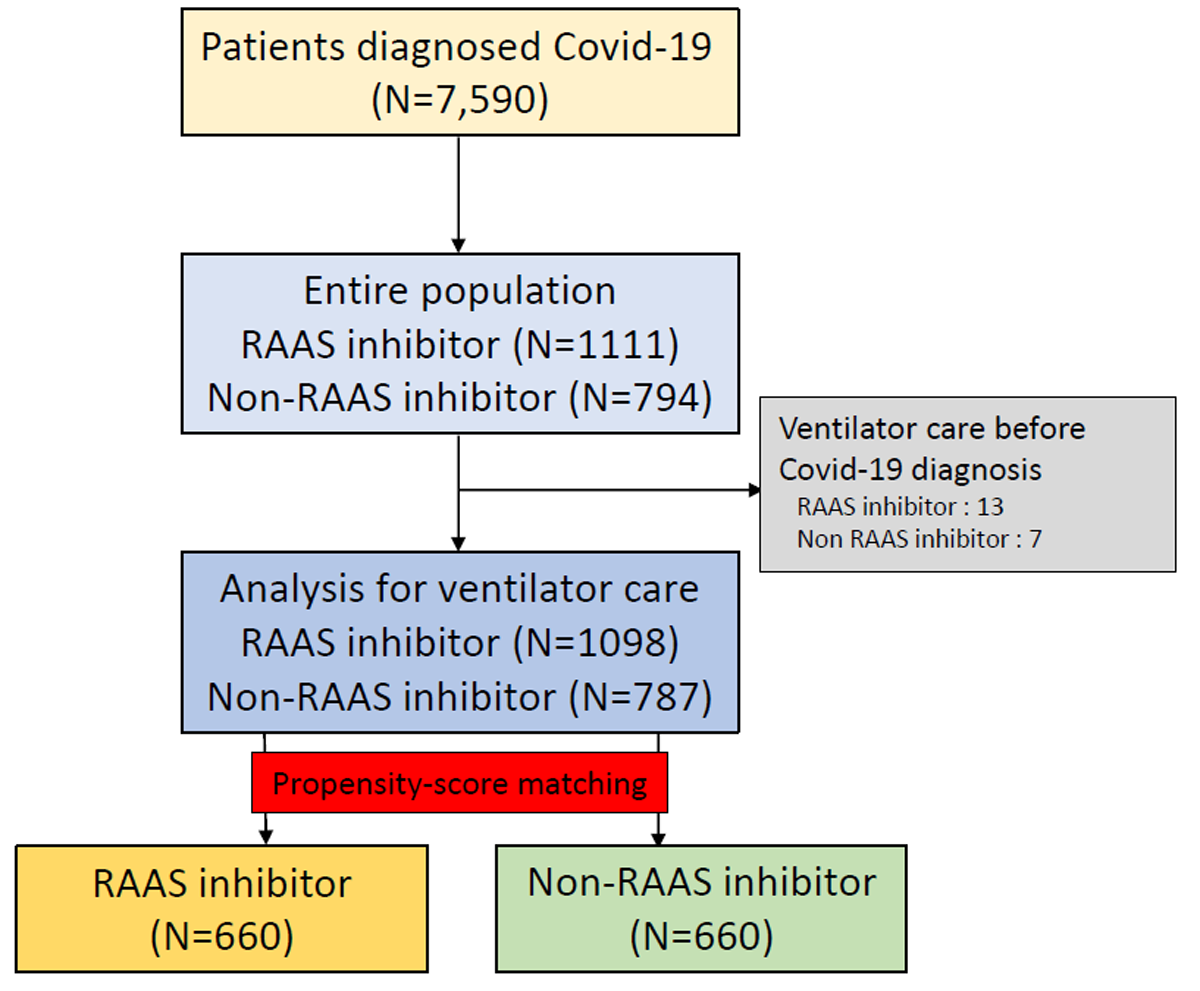

Supplement: S1 Fig — (TIF) [file pone.0248058.s001.tif]

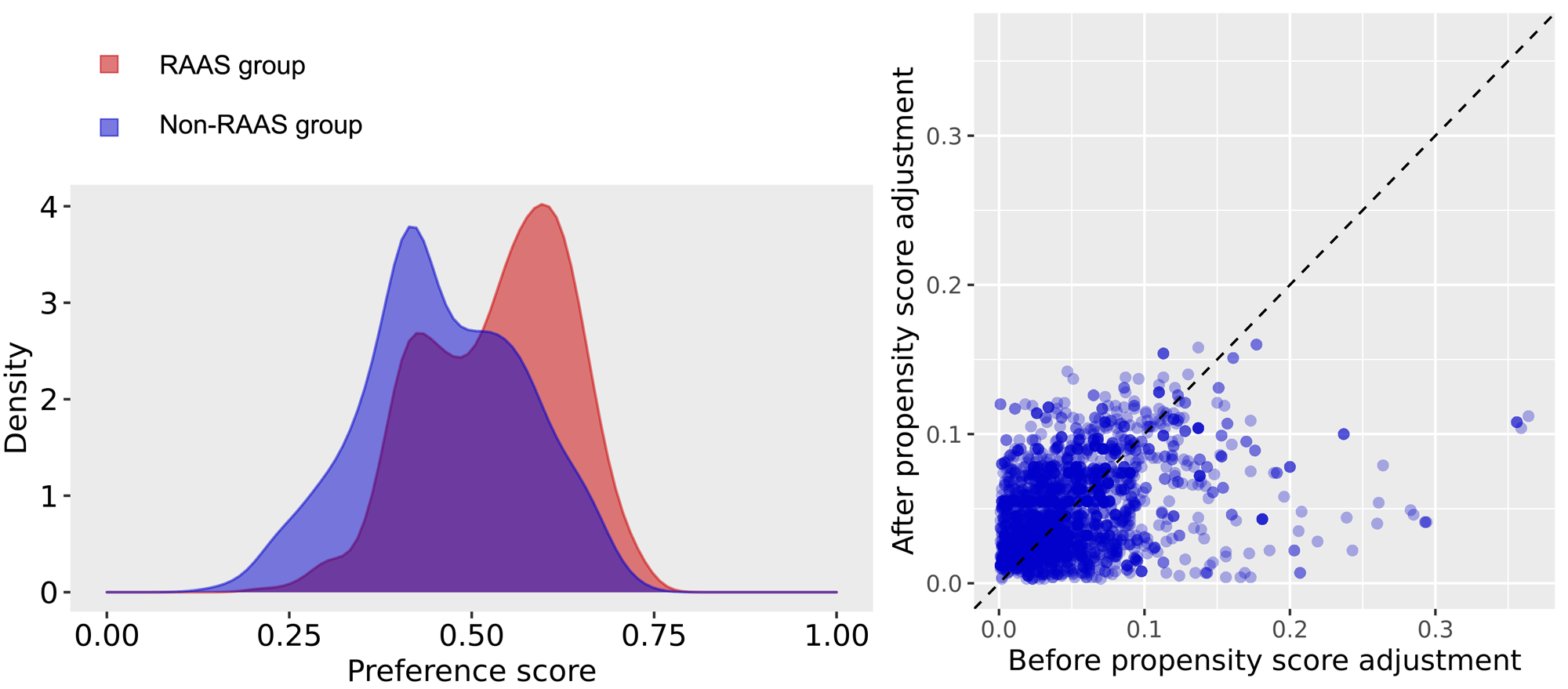

Supplement: S2 Fig — (TIF) [file pone.0248058.s002.tif]

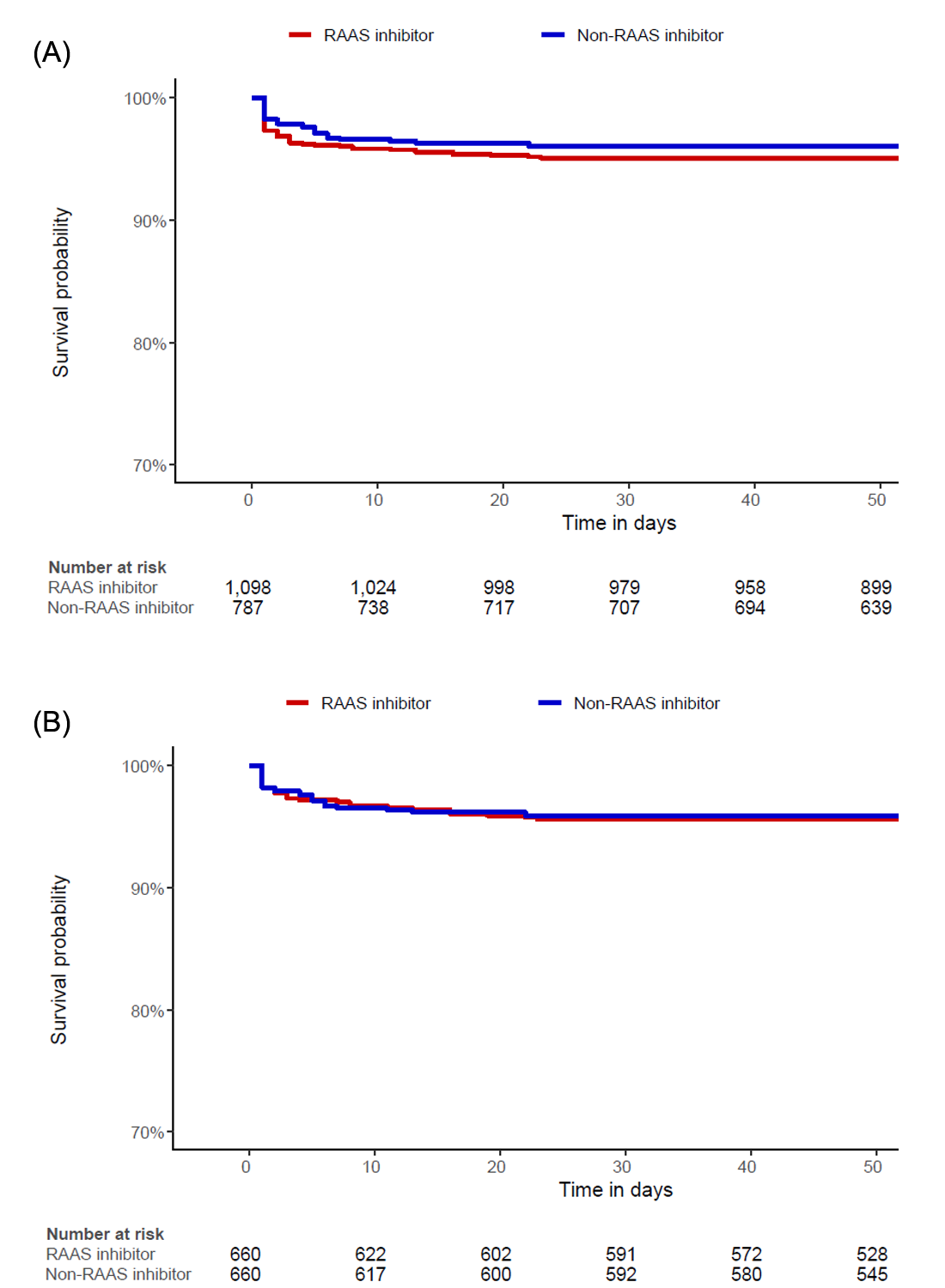

Supplement: S3 Fig — (TIF) [file pone.0248058.s003.tif]
