# Supplementary material for: Effect of renin-angiotensin-aldosterone system inhibitors on Covid-19 patients in Korea
Source: PLoS One. 2021 Mar 11;16(3):e0248058. doi: 10.1371/journal.pone.0248058 (PMC7951918; doi:10.1371/journal.pone.0248058)
Supplement: S1 Table — (DOCX) [file pone.0248058.s004.docx]

**Supplemental Table 1. Baseline Characteristics of Cohorts for Ventilator Care Comparison**

|  | **Before propensity score adjustment** | | | **After propensity score adjustment** | | |
| --- | --- | --- | --- | --- | --- | --- |
|  | **RAAS inhibitor** | **Non-RAAS inhibitor** | **SMD** | **RAAS inhibitor** | **Non-RAAS inhibitor** | **SMD** |
|  | **(N = 1,098)** | **(N = 787)** |  | **(N = 660)** | **(N = 660)** |  |
| **Age group** |  |  |  |  |  |  |
| 15-19 | 0.1 | 0.6 | -0.09 | 0.2 | 0.6 | -0.07 |
| 20-24 | 0.5 | 2.1 | -0.15 | 0.6 | 2 | -0.12 |
| 25-29 | 0.9 | 3.4 | -0.17 | 1.2 | 2.7 | -0.11 |
| 30-34 | 1 | 2.1 | -0.09 | 1.2 | 2 | -0.06 |
| 35-39 | 1.4 | 1.4 | 0 | 0.9 | 1.1 | -0.01 |
| 40-44 | 1.9 | 3.3 | -0.09 | 2.3 | 2.9 | -0.04 |
| 45-49 | 6.2 | 6.3 | 0 | 6.8 | 6.1 | 0.03 |
| 50-54 | 10.1 | 8.9 | 0.04 | 10.2 | 8.2 | 0.07 |
| 55-59 | 14.5 | 12 | 0.07 | 15.5 | 11.8 | 0.11 |
| 60-64 | 16.1 | 11.7 | 0.13 | 15.5 | 11.7 | 0.11 |
| 65-69 | 12.1 | 11.8 | 0.01 | 10.3 | 13 | -0.09 |
| 70-74 | 10.1 | 10.5 | -0.01 | 10.3 | 11.1 | -0.03 |
| 75-79 | 10.8 | 11.2 | -0.08 | 9.3 | 12 | -0.07 |
| 80-84 | 7.7 | 6.7 | 0.04 | 7.9 | 7.1 | 0.03 |
| 85-89 | 4.8 | 5 | -0.01 | 5.2 | 4.8 | 0.01 |
| 90-94 | 1.5 | 2.4 | -0.06 | 2.1 | 2.3 | -0.01 |
| 95-99 | 0.3 | 0.6 | -0.05 | 0.5 | 0.6 | -0.02 |
| **Sex: Female** | 55.9 | 55.4 | 0.01 | 55.6 | 54.5 | 0.02 |
| **Medical history** |  |  |  |  |  |  |
| Acute respiratory disease | 74.2 | 70.8 | 0.08 | 72.1 | 71.2 | 0.02 |
| Chronic liver disease | 9.6 | 8.6 | 0.04 | 8.8 | 9.7 | -0.03 |
| Chronic obstructive lung disease | 3.8 | 4.7 | -0.04 | 3.3 | 4.5 | -0.06 |
| Dementia | 11.7 | 16.9 | -0.15 | 13.3 | 15.9 | -0.07 |
| Depressive disorder | 19 | 27.2 | -0.2 | 23.9 | 21.5 | 0.06 |
| Diabetes mellitus | 41.6 | 28.2 | 0.28 | 29.8 | 32.1 | -0.05 |
| Gastroesophageal reflux disease | 44.8 | 44.7 | 0 | 41.4 | 45.3 | -0.08 |
| Gastrointestinal hemorrhage | 3.3 | 3.8 | -0.02 | 3.5 | 3.9 | -0.02 |
| Hyperlipidemia | 70.7 | 53.7 | 0.36 | 56.5 | 61.8 | -0.11 |
| Lesion of liver | 4 | 3 | 0.05 | 3.2 | 3.3 | -0.01 |
| Obesity | 0.3 | 0.1 | 0.03 | 0.5 | 0.2 | 0.06 |
| Osteoarthritis | 26 | 26.4 | -0.01 | 24.1 | 27.4 | -0.08 |
| Pneumonia | 51.8 | 49.5 | 0.05 | 49.4 | 50.8 | -0.03 |
| Psoriasis | 1.8 | 0.8 | 0.09 | 1.8 | 0.8 | 0.09 |
| Renal impairment | 6.8 | 4.7 | 0.09 | 5 | 5.2 | -0.01 |
| Rheumatoid arthritis | 4.7 | 4.3 | 0.02 | 4.4 | 4.8 | -0.02 |
| Schizophrenia | 3.7 | 6.7 | -0.14 | 5.2 | 5.3 | -0.01 |
| Urinary tract infectious disease | 7.5 | 9.4 | -0.07 | 7.3 | 8.8 | -0.06 |
| Viral hepatitis C | 0.5 | 1 | -0.05 | 0.3 | 1.1 | -0.09 |
| Visual system disorder | 49.3 | 49.6 | -0.01 | 46.7 | 50.8 | -0.08 |
| **Medical history: Cardiovascular disease** |  |  |  |  |  |  |
| Atrial fibrillation | 3.2 | 3.8 | -0.03 | 2.7 | 3.9 | -0.07 |
| Cerebrovascular disease | 9.2 | 6.8 | 0.09 | 8.6 | 7.6 | 0.04 |
| Coronary arteriosclerosis | 0.7 | 1.8 | -0.09 | 0.9 | 1.8 | -0.08 |
| Heart disease | 33.1 | 31.6 | 0.03 | 30.6 | 32.9 | -0.05 |
| Ischemic heart disease | 15.6 | 13.7 | 0.05 | 14.4 | 14.2 | 0 |
| Peripheral vascular disease | 17.2 | 15.7 | 0.04 | 14.2 | 17.7 | -0.1 |
| Pulmonary embolism | 21.3 | 17.8 | 0.09 | 21.4 | 19.5 | 0.04 |
| Venous thrombosis | 0.2 | 0.8 | -0.08 | 0.2 | 0.6 | -0.07 |
| **Medical history: Neoplasms** |  |  |  |  |  |  |
| Hematologic neoplasm | 0.1 | 0.3 | -0.04 | 0.2 | 0.3 | -0.03 |
| Malignant lymphoma | 7.7 | 8.6 | -0.03 | 7.1 | 8.6 | -0.06 |
| Malignant neoplastic disease | 0.6 | 0.4 | 0.04 | 0.5 | 0.3 | 0.03 |
| Malignant tumor of breast | 0.5 | 0.6 | -0.03 | 0.2 | 0.8 | -0.09 |
| Malignant tumor of colon | 0.6 | 0.4 | 0.04 | 0.8 | 0.3 | 0.06 |
| Malignant tumor of lung | 0.2 | 0.1 | 0.01 | 0.3 | 0.2 | 0.03 |
| Malignant tumor of urinary bladder | 1.2 | 1.5 | -0.03 | 0.9 | 1.7 | -0.07 |
| Primary malignant neoplasm of prostate | 0.1 | 0.3 | -0.04 | 0.2 | 0.3 | -0.03 |

Data are presented as %.

Abbreviations: RAAS, renin-angiotensin-aldosterone system; SMD, standardized mean difference
